# Transcutaneous Electrical Nerve Stimulation (TENS) in the Emergency Department for Pain Relief: A Preliminary Study of Feasibility and Efficacy

**DOI:** 10.5811/westjem.2018.7.38447

**Published:** 2018-08-09

**Authors:** Casey A. Grover, Mia Potter McKernan, Reb J.H. Close

**Affiliations:** * Community Hospital of the Monterey Peninsula, Department of Emergency Medicine, Monterey, California; † Community Hospital of the Monterey Peninsula, Community Health Innovations, Monterey, California

## Abstract

**Introduction:**

Given the high rates of opioid addiction and overdose in the United States, non-opioid means of treating pain are increasingly needed. Transcutaneous electrical nerve stimulation (TENS) therapy is an effective non-opioid modality for treating pain, but has not yet been routinely used in emergency department (ED) settings. In this study we asked the following questions: Are TENS units a feasible treatment for pain in the ED? How effective are TENS units for the management of pain in a general ED population?

**Methods:**

At our institution, we performed a pilot study using TENS units for pain. Patients in the ED were given, at the discretion of the ED provider, TENS units for the treatment of pain. Patients could be included for acute or chronic pain on whatever part of the body that was safe to use with TENS.

**Results:**

A chart review of patients receiving TENS units in the ED (n=110) revealed that TENS was useful in relieving pain, along with other treatments, in 99% of cases. When surveyed, 83% of patients reported a functional improvement while using the TENS, and 100% of patients would recommend a TENS unit to a family or friend. When surveyed, 100% of ED staff observed that TENS units were effective in treating pain for patients, and 97% would want to use them if they themselves were patients.

**Conclusion:**

Overall, in this small pilot study, TENS units appeared to be effective in our ED for reducing pain, when added to standard treatment. Additional studies are needed to determine which conditions are most responsive to TENS therapy, and the magnitude of pain reduction when used alone.

## INTRODUCTION

With the high incidence of addiction, overdose, and death from heavy prescribing of opioids in the United States,^1^–^3^ medical providers often try to avoid opioids but struggle with how to manage pain without using them. Transcutaneous electrical nerve stimulation (TENS) therapy is an effective non-opioid modality for treating pain,^4^–^16^ but it is not yet commonly used in emergency department (ED) settings. TENS works by a phenomenon called “gate control theory.”

There are multiple receptors in the periphery – pain, vibration, temperature, etc. – all of which transmit information to the brain via the spinal cord. The spinal cord fibers that transmit peripheral information cannot transmit information from multiple receptors simultaneously, and so the stimulation of multiple receptors at the same time results in decreased signal from each to the brain. TENS units, by providing a low-dose electrical current, stimulate vibration receptors, which when applied to an area having pain, reduces the transmission of painful stimuli to the brain.^4^ Additionally, when TENS units are repeatedly applied to an area, they increase the secretion of endogenous endorphins, reducing pain.^5^ As such, they are useful for the management of both acute and chronic pain.

The goal of this study was to evaluate the efficacy of TENS units for the treatment of pain in the ED. The study questions were as follows: 1) Are TENS units a feasible treatment for pain in the ED?; and 2) How effective are TENS units for the management of pain in a general ED population? The project was a pilot program in our ED, and our study reflects the program evaluation. To the best of our knowledge, this is the first program to use TENS units on a routine basis for pain management in the ED, and represents the first study of using TENS therapy in the ED.

## MATERIALS AND METHODS

This study was conducted at a suburban community hospital, with an annual ED census of approximately 56,000 yearly visits. The hospital developed a pilot study of using TENS units for pain management in the ED, and we report here the program evaluation of this project. The study was deemed a program evaluation by the hospital’s institutional review board committee, and therefore exempt from its approval.

When the project was designed, we chose to offer TENS therapy to our providers as a pain management option in our ED. We did not specify whether or not TENS units could be used as mono-therapy or in combination with standard treatment for painful conditions in our ED. While the overarching goal was to provide effective pain relief without the need for opioids, this project was a feasibility pilot study to see if TENS units could be used routinely in our ED for pain management, along with other treatments. ED staff were informed that we were conducting a pilot study of using TENS units in our ED for pain management, but were not informed how we would be collecting data.

We decided, when beginning the project, that we would collect data in three ways to evaluate the efficacy of our program: chart review of patients receiving TENS therapy; surveys of patient experience; and surveys of ED staff experience. Our study hypothesis was that as TENS units are effective in treating pain in multiple studies, TENS therapy for pain control in a general ED population would be, overall, effective in reducing pain. Our focus, given the preliminary nature of the study, was not to quantify the effect of pain relief, but rather to demonstrate that TENS could be used for pain control in an ED setting to support continuation of TENS therapy in our ED and provide preliminary positive study results to support additional, and more methodologically rigorous studies on the use of TENS units in the ED.

Patients were included in the study if they met the following inclusion criteria: age over 15 presenting with acute or chronic pain in any area of the body, and open to trying a TENS unit for pain control. Patients were chosen to receive a TENS unit at the discretion of the treating provider in the ED. Exclusion criteria, contraindications, and precautions for the use of a TENS unit are listed in Table 1.

**Population Health Research Capsule**

What do we already know about this issue?

In the setting of the U.S. opiate epidemic, non-opiate pain treatment is desirable. Transcutaneous electrical nerve stimulation (TENS) therapy is an effective non-opioid treatment for pain.

What was the research question?

Are TENS units a feasible treatment for pain in the emergency department (ED), and if so, how effective are they in treating pain in a general ED population?

What was the major finding of the study?

In this small pilot study, nearly all patients (99%) had relief of pain in the ED with TENS therapy.

How does this improve population health?

Using TENS units for pain relief in the ED could reduce the need for opioids, while still treating pain.

For those patients given a TENS unit, the patient’s name, age, medical record number, and email address (if available) were recorded for follow-up. The TENS units were applied by the treating provider to the area of greatest pain, guided by the following recommendations: First, when using the TENS unit, electrode pads should not be touching, and should be at least one inch apart. Second, the electrode pads should not be placed too far apart, as it reduces the efficacy of the therapy. Third, electrode pads should be placed surrounding the area of greatest pain, to allow for the electrical current to pass between the electrodes through the painful area.

Patients received instructions from the manufacturer and instructions written by ED staff after reviewing how to use the unit. Patients were treated with the TENS unit for 20–30 minutes. Patients could receive any other medication or treatment to manage pain as directed by the treating provider. Patients could adjust the settings of the unit by themselves, or with assistance from ED staff. At discharge, the patient took the TENS unit home with them for further use.

Our primary assessment of the efficacy of TENS units for pain management in our ED was chart review. An ED staff member performed a retrospective chart review of the cases in which a TENS unit was used, reviewing whether or not providers documented a response to the TENS unit, and whether or not the TENS unit was documented anywhere in the medical record as being helpful in relieving pain. We also recorded whether pain was acute or chronic, traumatic or atraumatic, and on which part of the body the TENS unit was applied. Acute pain was defined as less than three weeks in duration, while chronic pain was defined as three or more weeks in duration. Traumatic was defined as resulting from an acute traumatic injury, while atraumatic was defined as occurring in the absence of an injury.

Data collection was done using a pre-prepared collection spreadsheet in Microsoft Excel (Microsoft Corp., Redmond, Washington) in a standardized fashion. Secondarily, we also surveyed patients who received TENS units and surveyed ED staff on their experience with TENS units. We sent out an anonymous email-based survey (SurveyMonkey, San Mateo, CA) eliciting patient feedback within one week of the ED visit to all patients who listed an email address. After four months of using the units, all ED staff received an anonymous, email-based survey (SurveyMonkey) eliciting their feedback on how well the units worked. Copies of both surveys are available as supplemental files. Data analysis was done using Microsoft Excel (Microsoft Corp., Redmond, Washington).

The TENS unit used was made by AccuRelief (Compass Health Brands, Middleburg Heights, OH), and the model was “Dual Channel TENS Electrotherapy Pain Relief System.”

## RESULTS

Between September 2017 and February 2018, 110 patients in our ED were treated with TENS units for pain management. Of those, 70 (64%) were female, and the average age of treated patients was 49 years. Patients who received a TENS unit varied in age from 15 to 92 years.

In our chart review, in 97 out of 110 cases (88%) in which a TENS unit was used, the ED documentation reported how the patient responded to the TENS unit. In the remainder of cases, there was no documentation at all of the patient’s response to the TENS unit, merely that the patient had been treated with a unit.

In 96 out of 97 (99%) cases in which the response to the TENS unit was documented, the TENS unit improved the patient’s pain. Information about the type of pain being treated is reported in Table 2.

For patient surveys, we had email addresses for 60 patients out of the 110 total patients who received a TENS unit, and 14 out of 60 patients (23%) responded to our email-based survey. Of the responders, 80% reported that they used their TENS unit multiple times after their ED visit. Zero patients required an opioid for pain relief when using the TENS unit; 92% of patients reported that they would use a TENS unit in the future. Of the patients surveyed, 100% said they would recommend a TENS unit to a friend or family, and 83% reported a functional improvement while using a TENS unit. Average pain scores from the survey are reported in Table 3, along with 95% confidence intervals.

For staff surveys, 35 out of 132 ED staff (27%) responded to our survey: 20% of respondents were ED techs, 45% were nurses, 29% were physicians, and the remainder were a combination of scribes and physician assistants. The surveys indicated that 100% of all ED staff reported they had observed TENS units improving pain in the ED; 100% of all ED staff reported that patients liked TENS units, and 97% of ED staff reported that if they were a patient in the ED with a sprained back, they would want to receive treatment with a TENS unit. Furthermore, 100% of ED staff would recommend a TENS unit to a friend or family member.

## DISCUSSION

In our pilot study, TENS units appeared to improve pain in ED patients, when combined with standard ED therapy. Between chart review, patient responses, and ED staff observations, TENS units were observed to improve pain and be useful in the treatment of pain in the ED.

A TENS unit is an inexpensive and reusable device with few side effects for the management of pain; therefore, the device should be considered as a high-yield intervention for the treatment of pain in the ED, particularly in the setting of this nation’s opioid crisis and high rates of addiction. Our experience with this pilot study was that most ED providers are unfamiliar with the use of TENS units, creating a barrier to their use and implementation. In our ED, two providers (both are authors of this study) overcame this reluctance by championing the use of these devices, providing bedside teaching to all ED staff on how to use them, and developing protocols on their use. For departments considering using TENS units in their ED, one or more providers should consider taking the lead on implementing the project.

The authors are aware of the limitations of the study, as will be formally discussed below, but would like to highlight that this pilot study presents data that support our hypothesis that TENS therapy for pain control in a general ED population would be effective in reducing pain. As such, our hope is that the results of this study will encourage other institutions to consider similar pilot projects in their own institutions, and stimulate further, and more definitive, study on the topic. As mentioned earlier, in our ED TENS units were used in addition to standard treatment. Some patients received only a TENS unit, while others received multiple medications, including opioids in some cases.

Additionally, providers treated a wide array of complaints with TENS units, from humeral fractures and lumbar myofascial strains to chronic hip arthritis. As mentioned in the methodology section, our focus on the study was to prove that TENS units could be feasibly used in the ED setting for pain management, and that TENS units would be effective in reducing pain. Additional study to determine which types of pain and injuries respond best to TENS units would be useful to guide future implementation of programs for TENS therapy in the ED.

## LIMITATIONS

This manuscript represents a pilot study at a single hospital on the use of TENS units for pain control, and has several limitations. First, providers did not document the response to a TENS unit in all cases, and not all patients or ED providers responded to our survey. As such, it is possible that there is a bias toward a positive effect, given that not all providers and survey recipients responded. As our online patient survey was anonymous, we could not track which patients who provided email addresses responded or did not respond, so we were not able to obtain any information on response bias by type of pain or other characteristic.

Additionally, our study was not able to quantify the magnitude of pain relief with TENS as we did not quantify the amount of pain relief in our chart review. In the patient survey, the downward trending pain scores seen in the patients using TENS units who responded to our survey may represent the natural course of an acute myofascial injury such as a strain to improve over time rather than the effect of the TENS unit. We also chose to evaluate all complaints of pain, acute or chronic, in any part of the body. As such, we did not focus on which types of pain or locations of pain were most responsive to TENS treatment.

Additionally, as this study lacks blinding or randomization, there may have been bias due to staff over-documenting positive results to TENS therapy, in the hope of creating a positive study. While this is possible, it is unlikely. Even though ED staff were aware that we were conducting a study of the use of TENS units, they were not informed of our collection methods beyond a survey of staff experience. Lastly, as ED providers had a large amount of freedom in choosing who received a TENS unit, it is possible that there was a bias, when deciding which patients should receive a TENS unit, toward patients who had a favorable perspective regarding these devices. A randomized, controlled, and blinded study design would yield more accurate data.

## CONCLUSION

In this small pilot study of using TENS units in a community hospital ED, we found that these units were effective, when used with standard ED treatments, in reducing pain. Additional studies with more robust methodology are needed to confirm the utility of this treatment modality to support widespread adoption, and focus on what types and locations of pain are most responsive to treatment.

## Supplementary Information





## Figures and Tables

**Table 1:** Exclusion criteria/contraindications for the use of a transcutaneous electrical nerve stimulation (TENS) unit.

TENS unit cannot be placed over the eyes.
TENS unit electrodes cannot be placed on opposite sides of the head that would result in a transcerebral current.
TENS unit electrodes cannot be placed on the chest and back that would result in a transthoracic current.
TENS units cannot be placed on the anterior neck due to the possibility of a vasovagal event or laryngospasm.
TENS units cannot be placed internally.
TENS unit electrodes cannot be placed directly over the spinal column.
TENS unit electrodes should not be placed near any sort of implantable device (spinal stimulator, pacemaker, etc.) where current from the TENS would interfere with the device.
For pacemakers or pacemaker/defibrillators, a TENS unit must be placed at least six inches away from the pacemaker AND during initial TENS unit placement, the patient should be on a cardiac monitor to watch for any interference.
TENS units should not be used over the uterus in pregnant women.

**Table 2:** Information regarding type/location of pain in patients given a transcutaneous pain-relief unit (n=97).

	Total	Percent of total
Acute pain	54	55.7%
Chronic pain	43	44.3%
Traumatic pain	42	43.3%
Atraumatic pain	55	56.7%
Location of pain		
Back (thoracic/lumbar)	59	61.1%
Shoulder/clavicle	15	15.5%
Neck	8	8.2%
Flank/rib	6	6.2%
Hip	5	5.2%
Upper extremity	2	2.1%
Lower extremity	2	2.1%

**Table 3:** Pain scores from patient survey.

	Score is 0–10, 10 being most severe (with 95% CI)
Before using TENS unit	8.50 (7.52 – 9.48)
While using TENS unit	4.67 (3.51 – 5.89)
After ED visit, and after using TENS unit, on day of survey	2.58 (1.41 – 3.79)

*CI*, confidence interval; *TENS*, transcutaneous electrical nerve stimulation; *ED*, emergency department.

## References

[1] Gwira Baumblatt JA, Wiedman C, Dunn JR (2014). High-risk use by patients prescribed opioids for pain and its role in overdose deaths. JAMA Intern Med.

[2] Paulozzi LJ, Strickler GK, Kreiner PW (2015). Controlled Substance Prescribing Patterns – Prescription Behavior Surveillance System, Eight States, 2013. MMWR Surveill Summ.

[3] Guy GP, Zhang K, Bohm MK (2017). Vital Signs: Changes in Opioid Prescribing in the United States, 2006–2015. MMWR Morb Mortal Wkly Rep.

[4] Gozani SN (2016). Fixed site high-frequency transcutaneous electrical nerve stimulation for treatment of chronic low back and lower extremity pain. J Pain Res.

[5] Zhu Y, Feng Y, Peng L (2017). Effect of transcutaneous electrical nerve stimulation for pain control after total knee arthroplasty: a systematic review and meta analysis. J Rehabil Med.

[6] Santana LS, Gallo RB, Ferreira CH (2016). Transcutaneous electrical nerve stimulation (TENS) reduces pain and postpones the need for pharmacological analgesia during labour: a randomised trial. J Physiother.

[7] Bai HY, Bai HY, Yang ZQ (2017). Effect of transcutaneous electrical nerve stimulation therapy for the treatment of primary dysmenorrheal. Medicine (Baltimore).

[8] Mahure SA, Rokito AS, Kwon YW (2017). Transcutaneous electrical nerve stimulation for postoperative pain relief after arthroscopic rotator cuff repair: a prospective double-blinded randomized trial. J Shoulder Elbow Surg.

[9] Sharma N, Rekha K, Srinivasan JK (2017). Efficacy of transcutaneous electrical nerve stimulation in the treatment of chronic pelvic pain. J Midlife Health.

[10] Navarathnam RG, Wang IY, Thomas D (1984). Evaluation of the transcutaneous nerve stimulator for postoperative analgesia following cardiac surgery. Anaesth Intensive Care.

[11] Loh J, Gulati A (2015). The use of transcutaneous electrical nerve stimulation (TENS) in a major cancer center for the treatment of severe cancer-related pain and associated disability. Pain Med.

[12] Kong X, Gozani SN (2018). Effectiveness of fixed-site high-frequency transcutaneous electrical nerve stimulation in chronic pain: a large-scale observational study. J Pain Res.

[13] Jahangirifard A, Razavi M, Ahmadi ZH (2018). Effect of TENS on postoperative pain and pulmonary function in patients undergoing coronary artery bypass surgery. Pain Manag Nurs.

[14] Lison JF, Amer-Cuenca JJ, Piquer-Marti S (2017). Transcutaneous nerve stimulation for pain relief during office hysteroscopy: a randomized controlled trial. Obstet Gynecol.

[15] Eidy M, Fazel MR, Janzamini M (2016). Preemptive analgesic effects of transcutaneous electrical nerve stimulation (TENS) on postoperative pain: a randomized double-blind, placebo-controlled trial. Iran Red Crescent Med J.

[16] Mira TA, Giraldo PC, Yela DA (2015). Effectiveness of complementary pain treatment for women with deep endometriosis through transcutaneous electrical nerve stimulation (TENS): randomized controlled trial. Eur J Obstet Gynecol Reprod Biol.

