# Supplementary material for: Transcutaneous Electrical Nerve Stimulation (TENS) in the Emergency Department for Pain Relief: A Preliminary Study of Feasibility and Efficacy
Source: West J Emerg Med. 2018 Aug 9;19(5):872–6. doi: 10.5811/westjem.2018.7.38447 (PMC6123101; doi:10.5811/westjem.2018.7.38447)
Supplement: Supplementary file 1 [file wjem-19-872-s001.pdf]

CHOMP Emergency Department TENS Unit Survey

1. What day were you seen in the ER?

2. Where were you having pain?

- Neck
- Back
- Hip
- Thigh
- Lower Leg
- Knee
- Ankle
- Shoulder
- Upper arm
- Elbow
- Forearm
- Other (specify below)

Other (please specify)

3. Since you received the TENS unit, about how often have you used it?

- Once or twice
- Several times
- Daily
- Multiple times per day

4. What was your pain level, 1-10, before you received the TENS unit? (1 is minimal pain, 10 is the worst possible pain)

- 1
- 2
- 3
- 4
- 5
- 6
- 7
- 8
- 9
- 10

5. How would you rate your pain before you receive the TENS unit?

- ◯ Mild
- ◯ Moderate
- ◯ Severe

6. What is your pain level, 1-10, while using the TENS unit?

- ◯ 1
- ◯ 2
- ◯ 3
- ◯ 4
- ◯ 5
- ◯ 6
- ◯ 7
- ◯ 8
- ◯ 9
- ◯ 10

7. How would you rate your pain while using the TENS unit?

- ◯ Mild
- ◯ Moderate
- ◯ Severe

8. What is your pain level, 1-10, today?

- ◯ 1
- ◯ 2
- ◯ 3
- ◯ 4
- ◯ 5
- ◯ 6
- ◯ 7
- ◯ 8
- ◯ 9
- ◯ 10

9. How would you rate your pain today?

- ◯ Mild
- ◯ Moderate
- ◯ Severe

10. Are you taking any medications to help with pain, in addition to the TENS unit? (If yes, you can enter them in the comments field)

- Yes
- No

Other (please specify)

11. Are you taking opioid medication (painkillers such as oxycodone, Percocet, Norco, hydrocodone, tramadol, etc) to help with your pain in addition to the TENS unit?

- Yes
- No

12. Will you continue to use your TENS unit in the future?

- Yes
- No

13. Would you recommend a TENS unit to friends or family for the treatment of pain?

- Yes
- No

14. While using the TENS unit, did you have an improvement in your functional mobility

- Yes
- No

15. What is your name and date of birth? (This information will be used to review the diagnosis you were given to study how useful TENS units are). If you don't want to share that information, leave it blank
