# Supplementary material for: Transcutaneous Electrical Nerve Stimulation (TENS) in the Emergency Department for Pain Relief: A Preliminary Study of Feasibility and Efficacy
Source: West J Emerg Med. 2018 Aug 9;19(5):872–6. doi: 10.5811/westjem.2018.7.38447 (PMC6123101; doi:10.5811/westjem.2018.7.38447)
Supplement: Supplementary file 2 [file wjem-19-872-s002.pdf]

CHOMP ED Staff TENS Unit Survey

CHOMP ED Staff TENS Unit Survey

Hi all
We're trying to get feedback on how our use of TENS units is working in the ED.
Just in case you're not familiar with TENS units - they're the small devices that have wires attached to sticky electrode pads that we're putting on patients to help with pain. We've been using them for the past few months - and want to see what you think!
Thanks for your help!

1. What is your job in the ED?

- ○ Scribe
- ○ Tech
- ○ Nurse (RN or LVN)
- ○ Other (please specify)
- ○ UR
- ○ PA
- ○ Physician

2. Have you had an experience with a patient in our ED receiving a TENS unit?

- ○ Yes
- ○ No (if you answer no, skip to the end of the survey and hit "done"

3. Have you seen the use of a TENS unit improve pain in our ED?

- ○ Yes
- ○ No

4. How often have you seen a TENS unit improve pain when it is used?

- ○ All the time (100%)
- ○ Most of the time (over 50%)
- ○ Some of the time (less than 50%)
- ○ Rarely (most of the time it doesn't work)
- ○ Never (0%)
- ○ I don't have enough experience

5. Overall, do you feel that TENS units help relieve pain?

- ○ Yes
- ○ No

6. Overall, in your experience, do patients like using TENS units?

- ◯ Yes
- ◯ No

7. If you were a patient in our ED with a sprained back, would you want to try a TENS unit?

- ◯ Yes
- ◯ No

8. Would you - based on your ED experience with TENS units - recommend a TENS unit to a friend or family member?

- ◯ Yes
- ◯ No

9. If you didn’t already have one - has your experience in our ED with TENS units - made you consider using a TENS unit yourself?

- ◯ Yes
- ◯ No
- ◯ I already had one
- ◯ I haven’t had a need for a TENS unit

10. Do you have a TENS unit yourself?

- ◯ Yes
- ◯ No

11. If you have a TENS unit, do you find it useful to treat your own pain or injuries?

- ◯ Yes
- ◯ No
- ◯ I don’t have one

12. Do you have any suggestions on how we can improve the use of TENS units in our ED?

13. Do you have any other feedback, suggestions, opinions, or patient stories that you would like to share about the use of TENS units in our ED?
